# Enhanced inverse Faraday effect and time-dependent thermo-transmission in gold nanodisks

**DOI:** 10.1515/nanoph-2023-0777

**Published:** 2024-02-05

**Authors:** Alma K. González-Alcalde, Xinping Shi, Victor H. Ortiz, Ji Feng, Richard B. Wilson, Luat T. Vuong

**Affiliations:** University of California at Riverside, Riverside, CA, USA

**Keywords:** inverse Faraday effect, plasmonics, time-dependent transmission

## Abstract

Nonmagnetic media can be magnetized by light via processes referred to as an inverse Faraday effect (IFE). With nonmagnetic metal nanostructures, the IFE is dominated by the presence of light-induced solenoidal surface currents or plasmons with orbital angular momenta, whose properties depend on both the light and nanostructure geometry. Here, through a systematic study of gold nanodisks with different sizes, we demonstrate order-of-magnitude enhancement of the IFE compared to a bare gold film. Large IFE signals occur when light excites the dipolar plasmonic resonance of the gold nanodisk. We observe that the spectral response of the IFE signal mirrors the spectral response of time-dependent thermo-transmission signals. Our careful quantitative experimental measurements and analysis offer insight into the magnitude of IFE in plasmonic structures for compact, low-power, magneto-optic applications.

## 1 Introduction

Circularly polarized light can induce a nonequilibrium magnetization in a nonmagnetic material via the inverse Faraday effect (IFE). The earliest theoretical descriptions of the inverse Faraday effect were proposed for nonabsorbing media [1], [2], [3], [4]. The IFE magnetization generally refers to the induction of charge orbital motion by incident circularly polarized light [4], [5] or the excitation of electrons into electronic states with nonzero spin [6], [7], [8]. However, several theoretical studies predict that linearly polarized light [9], [10] and optical vortex and vector beams can also induce magnetic IFE moments [11], [12]. Our understanding of the IFE is currently being developed.

Prior theoretical and experimental studies have documented the phenomenology of the IFE in simple metals [3], [13], [14], [15], [16], [17]. The magnetic moment produced by circularly polarized light is proportional to the square of the electric-field amplitude [3], i.e., proportional to the light intensity. The magnetic moment can be monitored through time-resolved measurements of the off-diagonal terms of the optical susceptibility tensor of the metal [14], [17], [18]. The coherent motion of charges and the associated changes in the optical susceptibility persist for as long as the metal is excited with circularly polarized light [14]; once excitation with circularly polarized light stops, electron–electron and electron–phonon scattering randomize the angular momentum of the moving charge on a 10-fs time scale [14].

Knowledge of the IFE, particularly in thin films, is relevant to future computing, integrated photonic chips, and hybrid photonics–electronics hardware for applications such as ultrafast optical switching [19], magneto-optical data writing [20], [21], [22], [23], ultrafast microscopy [24], and spintronics [25], [26]. There is significant interest in enhancing the interaction strength between light and magnetic degrees of freedom in materials: ultrafast magneto-optic phenomena, like all-optical switching, requires electric field strengths on the order of 10^8^ V/m [27], which are nontrivial.

For thin-film applications, plasmonic nanostructures represent one of the most popular platforms for controlling, tuning, and enhancing light–matter interactions; excitation close to the plasmonic resonance may produce orders-of-magnitude enhancements in the local field [28], [29]. Several theoretical studies of IFE have predicted significant plasmonic enhancement of the magnetization induced by circularly polarized light [10], [16], [30], [31], [32], [33], [34]. The presence of the IFE can be inferred and observed indirectly via Lorentz-force mechanical effects, electrical potentials, or changes in optical spectra [5], [35], [36], [37], [38], [39], [40], [41]. However, there are only a few time-resolved measurements of the plasmonic IFE [42], [43]. Recent studies scrutinize the role of Lorentz forces in plasmon damping or IFE reversal [44], [45]. Since there are only a few quantitative experimental measurements of the plasmonic IFE, it remains unclear how and to what extent the IFE is enhanced in plasmonic structures, and what effect different types of plasmonic particle geometries and sizes have on the enhancement.

**Figure 1:**
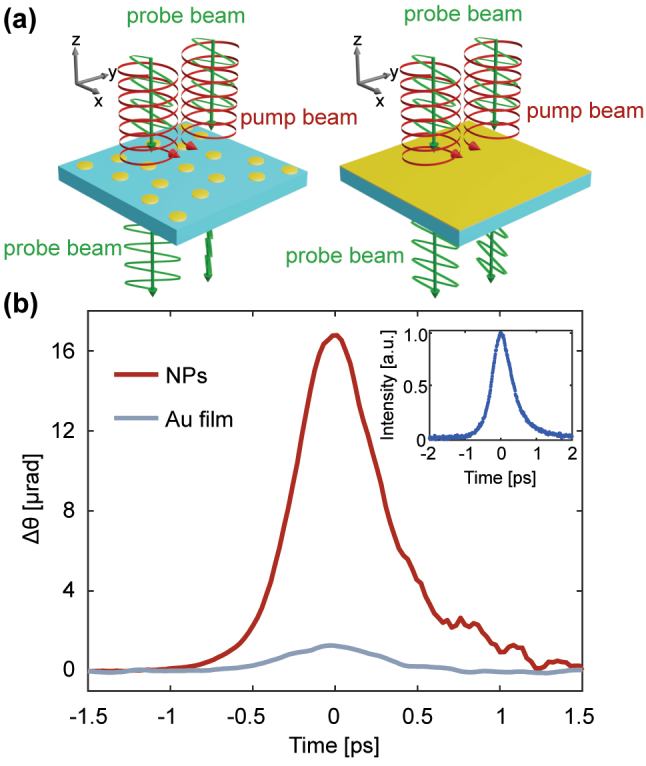
Inverse Faraday rotation. (a) Illustration of the light-induced rotation from the inverse Faraday effect in a nonmagnetic plasmonic monolayer of gold nanodisks and 20-nm thick gold film. (b) Measured rotation Δ*θ* = (*θ*_*RCP*_ − *θ*_*LCP*_)/2, where *RCP* and *LCP* are subscripts for right and left circular polarization. Inset: autocorrelation measurement of the laser pulse duration.

**Figure 2:**
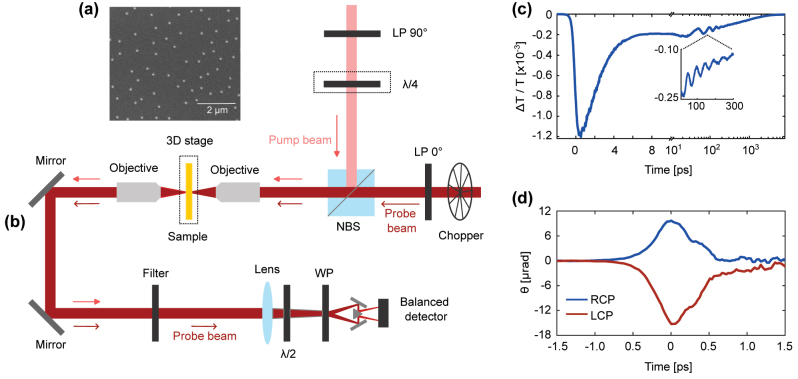
Time-resolved pump/probe experiments. (a) Scanning electron microscopy image of a Au nanodisk sample. The nanodisks in the image have 190-nm diameter and 24-nm height. (b) Experimental setup for the pump/probe experiments. The pump and probe wavelengths are 785 and 778 nm. The pump beam polarization is set to RCP or LCP by a combination of a linear polarizer (LP) and achromatic quarter-waveplate (*λ*/4) before a nonpolarizing beamsplitter (NBS). A 20X objective focuses both pump and probe on the sample. A half-waveplate (*λ*/2) before a Wollaston prism (WP) is rotated to balance two linear orthogonal polarization states with equal intensities at the detector. (c) Sample time-domain thermo-transmission (TDTT) measurement. The TDTT signal is shown on a linear scale from pump–probe delay times *t* = 0–10 ps and on a logarithmic scale from *t* = 10–300 ps. (d) Sample time-domain IFE (TD-IFE) measurements with RCP (blue line) and LCP (red line).

Here, we experimentally study the IFE and measure the Faraday rotation of gold nanodisks. To quantify the plasmonic enhancement, we compare the IFE signals from nanodisks to the IFE signals from a 20-nm thick gold film [Figure 1]. Additionally, we perform time domain thermo-transmission measurements of the nanodisks. The nanodisks that we study have different aspect ratios and, therefore, different longitudinal plasmonic resonance energies [46]. Most of the samples that we study are 190-nm diameter nanodisks [Figure 2(a)] with varied aspect ratios *h*/*D* between 0.13 and 0.21: this increase in aspect ratio leads to a blueshift in the plasmon resonance. Additionally, these nanodisks are considered to be large nanoparticles, where the scattering effects dominate over the absorption. We also characterize the IFE of nanodisks with diameters of 130 and 145 nm. When excited at the peak thermo-transmission wavelengths of the plasmonic resonance, we observe an order-of-magnitude enhancement in the IFE signals from nanodisks in comparison to a gold (Au) film. Finally, we observe that the spectral dependence of the IFE measurements mirrors the spectral dependence of time-domain thermo-transmission measurements.

## 2 Time-resolved pump/probe measurements

The experimental setup for time-domain inverse Faraday effect (TD-IFE) and time-domain thermo-transmission experiments utilize an 80-MHz Ti:Sapphire oscillator with 430-fs pulse durations and tunable wavelengths from 690 nm to 1050 nm (Mai-Tai) [Figure 2(b)]. We estimate the pulse duration from the autocorrelation measurement [inset of Figure 1(b)] by assuming both the pump and probe pulses have an intensity versus time described by the function $I=sech\left(2\ln\left(1+\sqrt{2}\right)\left(t/\tau \right)\right)$ , where *τ* is the duration. We perform two types of pump/probe experiments [Figure 2(c) and (d)]: time-domain thermo-transmission (TDTT) measurements that characterize the change in the sample transmission due to temperature or strain and time-domain IFE measurements that identify nonequilibrium changes of the transmitted probe-beam polarization caused by a right or left-circularly polarized pump-beam (RCP or LCP).

To prevent the pump beam from reaching the detector, the pump and probe beams are spectrally shifted from the Ti:Sapphire oscillator’s center wavelength of 783 nm. The pump passes through a long pass filter, and the probe beam passes through a short pass filter. After passing through the filters, the center wavelength of the beams is measured with a spectrometer (Ocean Optics). The pump and probe beams have wavelengths of 785 nm and 778 nm, respectively. A short-pass optical filter in front of the detector blocks the red-shifted pump beam.

For TDTT measurements, the experimental setup is the same as for TD-IFE measurements, but one of the inputs of the balanced detector is blocked. With this change, the signal becomes dominated by the change in the transmitted intensity, instead of the change in the polarization. The pump and probe average powers before the objective lens are kept constant in all of the experiments and set at 1.5 mW and 1.0 mW, respectively. The 20X objective lens has a transmittance of 0.7. The laser spot-size when focused on the sample is 3.9 μm. More details about the experimental setup can be found in Refs. [47], [48].

In Figure 2, we show a schematic of the experimental setup. In Figure 2(c), we show TDTT data taken from −2 ps to 8000 ps. The measurement allows us to monitor changes in transmission of the probe beam due to temperature and acoustic vibrations. The TDTR data from 10 ps to 300 ps identifies acoustic vibrations on the sample and is also used to determine the nanodisks’ dimensions in the region where the laser is focused. The physics of the nanodisk acoustic vibrations are described in detail in Ref. [49].

In Figure 2(d), we present TD-IFE data taken from −1.5 ps to 1.5 ps. To determine the circularly polarized light-induced rotation (Δ*θ*), we perform measurements for RCP (blue solid line) and LCP (red solid line). The circularly polarized light-induced rotation is Δ*θ* = (*θ*_*RCP*_ − *θ*_*LCP*_)/2. For the experimental setup shown in Figure 2, the TD-IFE signal is a measure of the real part of the Faraday angle, *i.e.*, polarization rotation. The curves have a hyperbolic secant shape that match the autocorrelation measurement of the pump and probe pulse duration. The temporal resolution of our experiments is not sufficient to resolve the lag between when the pump beam turns on and the Faraday rotation starts, which is expected to take 10 fs [16]. Similarly, we cannot resolve the lag between when the pump beam turns off, and the Faraday rotation ceases, which is expected to occur on a 10 fs time-scale due to momentum relaxation of excited electrons [14]. The sign of the polarization rotation is opposite for LCP versus RCP pump excitation. There is an asymmetry in the LCP versus RCP signals [Figure 2(d)] caused by a background TDTT signal. The background is also the cause of a small peak time-domain IFE signals in Figure 2(d) near 0.3 ps. The background is independent of polarization and so does not affect Δ*θ*. The Supplementary Information provides additional discussion of the background.

## 3 Sample synthesis and characterization

To control the frequency of the localized surface plasmon resonance (LSPR), we synthesize nanodisks with varied aspect ratio (AR = *h*/*D*) [50], [51]. For disks with a low aspect ratio, the LSPR frequency in the quasistatic limit is

(1) $$\omega =\frac{\pi h}{4D}\omega_{p}^{2}-\gamma^{2}$$

where *ω*_*p*_ is the plasma frequency and *γ* is the damping term. Smaller-AR nanodisks have resonances at lower frequencies, while larger-AR nanodisks are associated with resonances at higher frequencies.

The Au nanodisks were fabricated on a sapphire substrate by using a hole-mask colloidal lithography technique [52]. Sapphire substrates were cleaned by sonicating in acetone, ethanol, and water for 10 min each and then blow-dried. A sacrificial layer of poly(methyl methacrylate) (PMMA, MicroChem 495 A5) was spin-coated onto the precleaned sapphire substrates at 4000 rpm for 60 s and baked at 170 °C for 10 min to evaporate the solvent. The surface was made hydrophilic by a short period of oxygen plasma treatment (EMS 1050X Plasma Asher) at 100 W for 20 s. The coated substrate was immersed in a water solution of poly(diallyldimethylammonium chloride) (PDDA, Sigma Aldrich, 0.2 wt%) for surface modification with positive charges, rinsed with water thoroughly, and then blow-dried. The modified substrate was immersed in a water suspension of well-dispersed polystyrene (PS) beads (0.05 mg/mL, Bangs laboratories, PC02008) for 4 min to load the nanoparticles, rinsed with water thoroughly, and blow-dried. After that, 5 nm of Ti and 30 nm of Au (e-beam evaporation, Temescal BJD 1800) was deposited on the substrate as an etch mask, followed by sonicating in water for 30 s to remove the PS beads. The exposed PMMA layer was fully removed by reactive ion etching (STS Reactive Ion Etcher, 200 mTorr, 100 W, O2, 5 min). The Au nanodisks (with 5-nm Ti adhesion layer) were deposited on the sapphire substrate by E-beam evaporation, followed by a lift-off step (sonicate in acetone for 2 min). The final products were obtained after depositing a 3-nm thick layer of Al_2_O_3_ and annealed at 400 °C for 15 min.

The nanodisks were synthesized out of Au for several reasons. The small intrinsic absorption of Au, weak electron–phonon coupling [53], and lack of chemical reactivity make Au the plasmonic material of choice for many applications [54], [55], [56], [57]. Interband transitions between d-bands and the Fermi-level can have a dramatic effect on both the magneto-optical Kerr effect [48] and nonmagnetic Kerr effect [58]. However, such interband transitions in Au require photon energies above 2 eV [59] and so do not affect our experiments on disks in the near-infrared. (Such interband transitions would matter if we were studying spheres because they have plasmonic resonances at higher energies than disks). We would expect similar behaviors from plasmonic disks made from free-electron-like metals without interband transitions in the near-infrared, e.g., Ag or Cu.

We characterize the samples with scanning electron microscopy (SEM) and atomic force microscopy (AFM). We also use TDTT measurements to estimate the diameters of the nanodisk through the acoustic modes. Estimates of the nanodisk diameter are comparable given both acoustic frequency and microscopy images. We estimate that the nanodisk samples have diameters of *D* = {190, 190, 200, 190, 200} nm and heights of *h* = {24, 27, 30, 33, 41} nm. We estimate the nanodisks aspect ratios (AR = *h*/*D*) AR = {0.13, 0.14, 0.15, 0.17, 0.21}. The samples have an average of {1.6, 1.2, 1.3, 0.9, 1.5} nanodisks per μm^2^. The role of sample nanodisk density is discussed in Section 5.

**Figure 3:**
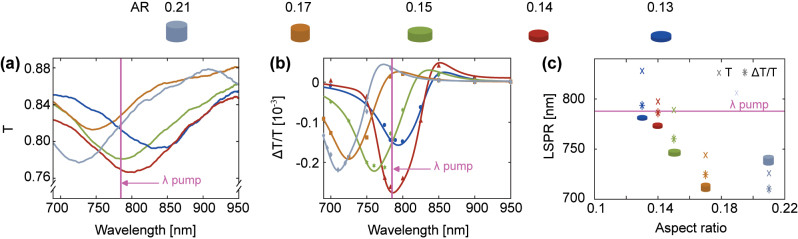
Spectral characterization of the samples. (a) Static transmission spectra. (b) Time-domain thermo-transmission spectra collected at a pump–probe delay time of 10 ps. (c) Sample resonance wavelength with static transmission (crosses) and TDTT measurements (asterisks).

We perform both static transmission and TDTT spectral characterizations of the 200-nm diameter nanodisks. We measure the static transmission spectra with direct illumination and detection. The static transmission spectra are normalized by the spectra of the lamp. The white light emission is unpolarized and the beam spot is 1 mm in diameter. The results are presented in Figure 3(a).

In addition to the ≈200-nm nanodisk samples described above, we prepared an additional set of samples with target diameters of 130 and 145-nm and aspect ratios that vary between 0.08 and 0.21. The samples were synthesized in a similar way as described above, but with the following changes. We used PMMA A4 as the sacrificial layer (Kayalu 495 A4). After spin-coating, the substrate was immersed in a water suspension of well-dispersed polystyrene (PS) beads (0.05 mg/mL, Bangs laboratories, PC02005, PC 02006). Due to the usage of different PMMA, the thickness of the PMMA layer changed, which required a change in the reactive ion etching time. In this new set of samples, the exposed PMMA layer was removed by reactive ion etching for 3 min. We performed static spectral characterizations, AFM, and SEM on all samples. We only performed pump/probe measurements on two of the samples: the 130-nm diameter disks with AR = 0.12 and the 145-nm diameter disks with AR = 0.14. These two samples had a transmission minima at the 785-nm wavelength of our laser. SEM images of the samples identified the density of 130-nm and 145-nm diameter nanodisks as 0.7 and 0.6 disks per μm^2^, respectively.

To collect the TDTT spectra, TDTT measurements versus wavelength are performed at a fixed time-delay of 10 ps. To perform the measurements, we modify the optical setup shown in Figure 2. The main changes regarding the optical setup are the following: (1) pump and probe beam are not collinear (there is a small angle between the pump and probe), (2) the pump beam is made vertically polarized by removing the *λ*/4 plate in Figure 2(b), (3) all optical filters that blue-shift the probe beam and red-shift the pump beam are removed, (4) an aperture is added to the detection line to spatially filter the pump beam, and (5) a linear polarizer (LP) is added to the detection line to filter the vertically polarized pump beam. The results of the TDTT spectra are presented in Figure 3(b).

As expected from Eq. (1), there is a blue shift in both static transmission peaks and TDTT spectra peaks as the nanodisk AR increases. For the static transmission measurements, the 785-nm wavelength pump is close to transmission extrema for the nanoparticles with an AR of 0.14 (red line) and 0.15 (green line) [Figure 3(a)]. Alternatively, the TDTT spectra extrema is closest to the pump wavelength for the samples with an AR of 0.14 (red line) and 0.13 (blue line) [Figure 3(b)]. In other words, the TDTT spectra are blue-shifted in comparison with respect to the static transmission spectra [Figure 3(c)]. In our discussion below, we label the TDTT LSPR as the extrema in the TDTT spectra. That the TDTT LSPR is blue-shifted from the static transmission spectra LSPR can be explained in terms of the time-dependent, temperature-induced change of the gold nanodisk dielectric constant by the pump beam [60], [61]. The TDTT spectra are a measure of the difference in the transmission spectra at an elevated versus ambient temperature. The sample transient transmission spectra are connected to the change in the dielectric function by

(2) $$\Delta Q_{\mathrm{ext}}\left(\lambda ,t\right)=\frac{\partial Q_{\mathrm{ext}}}{\partial \varepsilon_{r}}\frac{d\varepsilon_{r}}{dt}+\frac{\partial Q_{\mathrm{ext}}}{\partial \varepsilon_{i}}\frac{d\varepsilon_{i}}{dt},$$

where *Q*_*ext*_ is the extinction cross section, *λ* is the wavelength, *t* is the temperature, and *ɛ*_*r*_ and *ɛ*_*i*_ are real and imaginary parts of the dielectric function. According to Eq. (2), the spectral position of the dipolar resonance is temperature dependent. The TDTT peak blue shifts upon heating and can gain a positive side-wing [Figure 3(b) and (c)]. The TDTT spectra are narrower than the static transmission spectra. This is due to the fact that the transient transmission spectra carry terms associated with the derivative of the extinction cross section [Eq. (2)].

## 4 Results

**Figure 4:**
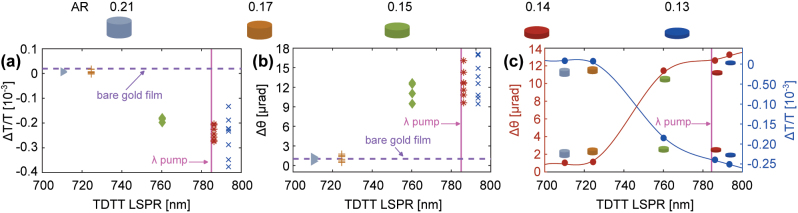
Time-domain pump/probe measurements of the samples. (a) Time-domain thermo-transmission (TDTT) measurements at 10 ps. (b) Inverse Faraday rotation measurements at 0 ps. The measurements are for Au nanodisk monolayers with different aspect ratios and a 20-nm bare gold film used as a reference. The wavelength of the pump beam is 785 nm. Each marker is a measurement in a different sample location. (c) Average of the inverse Faraday rotation measurements at 0 ps and TDTT measurements at 10 ps.

Samples with TDTT LSPR near the laser wavelength have order-of-magnitude enhancements in the TD-IFE and TDTT signals [Figure 4]. The TDTT (Δ*T*/*T*) data in Figure 4(a) are collected at a pump/probe time delay of 10 ps. The IFE rotation (Δ*θ*) in Figure 4(b) is the maximum Faraday rotation, which we observe at 0 ps delay time. For both Figure 4(a) and (b), we plot the data as a function of TDTT LSPR position [Figure 3(b) and (c)]. Each marker in Figure 4(a) and (b) represents a measurement at a different location on the sample.

For samples with 0.13 ≤ AR ≤0.15 , the Δ*T*/*T* is much larger compared with a 20-nm thick gold film (purple line). Samples with AR of 0.13 (blue marks) and 0.14 (red marks) have the largest (Δ*T*/*T*) values [4(a)]. This is because for both samples, the pump laser is close to the TDTT LSPR. On the other hand, we note a decrease in Δ*T*/*T* as the AR increases and indeed a change of sign in Δ*T*/*T* for AR ≥0.17 .

Samples with lower AR produce larger inverse Faraday rotations [Figure 4(b)]. The IFE enhancement is ∼17× (AR = 0.13), ∼16× (AR = 0.14), ∼12.5× (AR = 0.15), ∼1.8× (AR = 0.17), and ∼1.3× (AR = 0.21) larger than the IFE rotation from the 20-nm thick Au film. As expected, the IFE enhancement is largest for nanodisks whose TDTT LSPR is closest to the laser wavelength of 783 nm.

We observe a correlation between the TDTT and IFE measurements. This is shown in Figure 4(c) where we plot the average of all measurements reported in Figure 4(a) and (b). We observe that higher Δ*T*/*T* signals are associated with higher rotations Δ*θ*.

**Figure 5:**
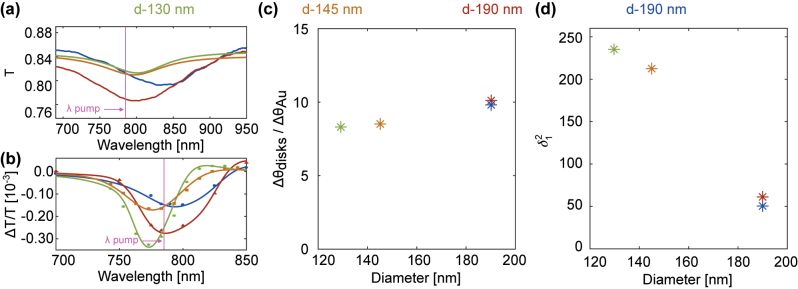
Inverse Faraday rotation measurements for nanodisks with different diameters and aspect ratios. (a) Static transmission spectra for samples with different diameters and areal densities. (b) Corresponding TDTT spectra at 10 ps: the TDTT LSPR resonances are close to the pump wavelength. (c) Measured Faraday rotation for these samples relative to Au thin-film. (d) Enhancement of the measured rotation *δ*_1_, which accounts for differences in the optical coverage area, see Section 5.

To further explore plasmonic enhancement of the IFE, we perform TD-IFE measurements of 130-nm and 145-nm diameter nanodisks with AR of 0.12 and 0.14. Both of the 130 and 145 nm-diameter nanodisk samples have a minima in the static transmission spectra [Figure 5(a)] near 800 nm and a TDTT LSPR wavelength [Figure 5(b)] close to the pump laser (773-nm and 775-nm peak values for 130- and 145-nm diameter nanodisks, respectively). The 130 and 145-nm diameter samples have areal densities that are one-sixth and one-fifth of the 190-nm disks. The IFE rotation measurements relative to the Au thin-film are summarized in Figure 5(c). We include the average IFE enhancement relative to the Au thin-film measured for the 0.13-AR and 0.14-AR, 190-nm diameter disks. The IFE enhancement with the smaller nanodisks is comparable to that of the 190-nm diameter disks even though these lower-density samples have half the extinction of the 190-nm diameter disk samples. We plot the nanodisk-to-thin-film rotation accounting for the areal density $\delta_{1}^{2}$ in Figure 5(d). The calculation of *δ*_1_ is described in detail and discussed in Section 5.

## 5 Discussion

### 5.1 Comparison with prior measurements

In response to ≈0.5 TW/m^2^ of CP excitation, the 190-nm diameter disks with 24-nm thicknesses display approximately 12-μrad of Faraday rotation or ≈24 μrad rotation per TW/m^2^. Despite significant differences in experimental geometry, this is in reasonable agreement with the results of Cheng et al. [42], who performed pump/probe IFE measurements on 2-mm thick colloidal suspensions of 100-nm diameter Au nanoparticles and reported a Faraday rotation of ≈30 μrad per TW/m^2^.

The light-induced nonequilibrium rotation on Au nanodisks in our study is significant; however, it is small when compared to materials that are magnetic at equilibrium. Ferromagnetic metals have Kerr rotations on the order of mrad or 100× greater than we observe from the nanodisks. The μrad-scale polarization rotation in the present experiments is similar in magnitude to the Kerr rotations caused by a few A/m of spin accumulation in a Au thin-film [48].

### 5.2 Magnetization versus Faraday rotation

To understand the magnitude of the magnetic moment induced in the disks, we now frame the relationship between Faraday rotation and magnetism. Consider *K* as a material-specific constant where the rotation is *θ* = *KM*_*ind*_. The range of reported values for *K* in bulk materials is between 4 and 40 nrad per A/m [48], [62]. In our experiments, we expect plasmonic enhancement of the induced magnetization: *M*_*disk*_ = *δ*_1_ *M*_*Au*_, where *M*_*disk*_ is the magnetization induced in the Au nanodisks and *M*_*Au*_ is the magnetization induced in the Au thin-film, and *δ*_1_ > 1 represents an Au geometric nanostructure plasmonic enhancement factor. The induced magnetization *M*_*ind*_ is expected to be proportional to the square of the electric field, and this local field is enhanced by plasmonic nanostructures. We are primarily interested in *δ*_1_, *i.e.*, how much the plasmonic resonance increases the CP light–induced magnetization.

Now, we also expect a plasmonic enhancement of *K* that occurs when we probe the nanodisk samples: *K*_*disk*_ = *δ*_2_ *K*_*Au*_. Based on numerous studies or Kerr and Faraday rotations in plasmonic structures such as [63], we also expect *δ*_2_ > 1. To crudely estimate the order of magnitude of *δ*_1_ from our experiments, we assume *δ*_1_ = *δ*_2_, which is reasonable since both factors describe plasmonic enhancements of the disk’s magneto-optical properties. (As shown below, this assumption yields the conclusion that *δ*_2_ is on the order of 10, which is in good agreement with prior studies of the Faraday effect in similar disks [40]). Then, we expect the enhancement factor in our TD-IFE experiments to be $\theta_{\mathrm{disk}}=\delta_{1}^{2}\theta_{Au}$ . This means (1) the rotation measurements from the plasmonically enhanced magnetization are also plasmonically enhanced and (2) induced magnetizations that are smaller or larger than what we infer could be possible.

### 5.3 Effect of areal coverage

Due to the sparse nanodisk coverage in our samples, most of the probe light incident on our samples is transmitted without interacting with the Au nanodisks. Our 190-nm diameter disks with AR = 0.13–0.14 have a surface coverage of 4–5 percent, respectively. Meanwhile, our 130- and 145-nm diameter disks in Figure 5 have surface coverage of only of ≈1 percent. On the other hand, Au thin films have 100-percent surface coverage.

The lower areal coverage means that the measured Faraday rotations underestimate the actual CP light–induced magnetization. The measured Faraday rotation should be scaled by a numerical factor 1/*C* related to the coverage area, where *C* < 1. We can estimate *C* based on the optical cross section of the disks. The measured attenuation of the nanodisks at the laser’s wavelength (Figure 3(a)) is ∼4× larger than the areal coverage of the nanodisks. Therefore, we expect the optical cross section of the nanodisks to be ∼4× the physical coverage area. (This is in reasonable agreement with reports in the literature for nanodisks with diameters between 100 and 200 nm [64], [65]). We estimate the optical coverage of the AR = 0.13 and 0.14 disks with 200-nm diameter to be *C* ≈ 0.16 and 0.2. Similarly, the optical coverage for the 130 and 145-nm diameter disks will be *C* ≈ 0.04.

Accounting for these differences in optical coverage, the plasmon enhancement in the CP light–induced magnetism is $\delta_{1}^{2}∼\Delta \theta_{\mathrm{disk}}/\Delta \theta_{Au}/C$ . Therefore, $\delta_{1}^{2}\approx 60$ and $\delta_{1}^{2}\approx 50$ in the 0.13-AR and 0.14-AR, 200-nm diameter nanodisks, respectively. In the 130 and 145-nm diameter disks, $\delta_{1}^{2}\approx 240$ and $\delta_{1}^{2}\approx 210$ . [The values of Δ*θ*_*disk*_/Δ*θ*_*Au*_ are taken from Figure 5(c)]. So, a reasonable estimate for the IFE enhancement in our experiments is *δ*_1_ ≈ 7 for 190-nm diameter disks, and *δ*_1_ ≈ 15 and *δ*_1_ ≈ 15 for the 130- and 145-nm disks.

In summary, after accounting for differences in optical coverage (*C*) and Faraday rotation per moment (*K*_*disk*_), our best estimate from our experimental data is that the magnetization induced by CP light is approximately one order of magnitude larger in Au nanodisks than a 20-nm thick Au film. Having established this experimental evidence, we now turn our attention to comparison with theoretical predictions for IFE in bulk Au and nanoparticles excited at resonance with CP light.

### 5.4 Comparison to theory

The light-induced magnetization can be modeled as an interaction between the illuminating light and the light-induced charge-density oscillations [3]. Hertel predicts that for uniform light illumination of a solid-state bulk material where the charge conductivity follows a simple Drude model [3], the induced magnetization is

(3) $$M=\frac{e\epsilon_{0}\omega_{p}^{2}}{4m\omega^{3}}|E{|}^{2},$$

where **E** is the electric-field amplitude and *ω* is the frequency of the circularly polarized light, *ω*_*p*_ is the plasmon frequency of Au, *e* is the charge of an electron, *m* is the free electron mass, and *ϵ*_0_ is the permittivity of free space. For Au, Hertel’s model predicts a magnetic moment of ≈8 A/m per TW/m^2^, or $\approx 1.5\times 10^{-5}\mu_{B}$ per atom per TW/m^2^. (Hertel’s model neglects the effect of spin-moments induced by interband transitions. However, Mishra and Coh predict that the spin contribution to the IFE due to interband absorption ranges between only 10^−7^ and 10^−6^ *μ*_*B*_ per atom per TW/m^2^ for visible light [8]). To our knowledge, quantitative measurements of the magnetization induced by circularly polarized light are not available for Au. However, the result from Eq. (3) is similar in magnitude to what is observed for simple ferromagnetic metals. Choi et al. report that excitation with 10 TW/m^2^ of circularly polarized light induces a 50–60 A/m magnetization in 10-nm thick Co, Fe, and Ni films capped with 2-nm layers of Au [22].

The framework described above can also be used to predict the IFE in nanoparticles driven at a plasmonic resonance that enhances local electric fields. The effect of the nanoparticle resonance in Eq. (3) is to replace the factor of 1/*ω* ^3^ in Hertel’s expression with a factor of 1/*γ* ^2^ *ω* [16],

(4) $$M=\frac{e\epsilon_{0}\omega_{p}^{2}}{4m\omega \gamma^{2}}|E{|}^{2},$$

where *γ* is an effective plasmon damping rate from a Drude model [16]. Eq. (4) predicts that the IFE is enhanced in a nanoparticle by the square of its quality factor, (*ω*/*γ*)^2^ [16], [32]. For our Au nanodisk samples, the width of the absorption peak in the static transmission spectra ranges between 100 and 200 nm [Figures 3(a) and 5(a)], corresponding to a quality factor <10.

Quantum mechanical based theories, which model dynamics without making a Drude-like free-electron assumption, predict somewhat different magnitude for the IFE than the classical models described above [16], [32]. Hurst et al. predict that a 1-nm radius sphere with 510 TW/m^2^-intensity circularly polarized light induces a total magnetic moment of $\approx 10\mu_{B}$ or $\approx 8\times 10^{-5}\mu_{B}$ per atom per TW/m^2^ [32]. Sinha-Roy et al. report that a 1-nm radius Au sphere with CP light of 13 TW/m^2^-intensity induces a total magnetic moment of $\approx 2\mu_{B}$ or $\approx 6\times 10^{-4}\mu_{B}$ per atom per TW/m^2^ [16]. We note that these predictions of quantum mechanical models may not be directly comparable to our values for *δ*_1_, since they model a much smaller volume of atoms.

The large *δ*_1_ that we measure in disks with small diameter is notable. In a parallel work, we theoretically show that the Faraday rotation is largely enhanced at the nanodisk LSPR when the extinction efficiency is dominated by absorption. For a single nanodisk, the effect of light scattering is to dampen the free-electrons oscillations, increase the linewidth, and significantly reduce the rotation efficiency [66]. We expect that similar effects may lead to a larger IFE in disks with smaller diameter and flatter nanodisks than wider, taller nanodisks with the same LSPR wavelength [Figure 5(d)].

## 6 Conclusions

In summary, we perform a systematic experimental study of the IFE in monolayers composed of gold nanodisks with different aspect ratios. Through pump–probe experiments, we measure that the inverse Faraday rotation is enhanced if the pump wavelength is close to the peak TDTT LSPR, which is blue-shifted from the static transmission measurement of the LSPR. We measure Faraday rotations approximately 10× larger compared to 20-nm thick bare gold film. The Faraday rotation per intensity with monolayers of nanodisks with <50-nm thicknesses are comparable to that reported in Ref. [42] with 2-mm thick colloidal suspensions. When further accounting for the measurement and optical cross section, the Faraday rotation per intensity per area is estimated to be as much as 240× larger than those observed from a gold film.

## Supplementary Material

Supplementary Material Details
