# Supplementary material for: Enhanced inverse Faraday effect and time-dependent thermo-transmission in gold nanodisks
Source: Nanophotonics. 2024 Feb 5;13(11):1993–2002. doi: 10.1515/nanoph-2023-0777 (PMC11501565; doi:10.1515/nanoph-2023-0777)
Supplement: Supplementary file 1 — Supplementary Material Details [file j_nanoph-2023-0777_suppl_001.pdf]

Supplementary Information

Photoexcitation of the sample with circularly polarized light causes two changes in the sample's optical properties.

(1) Energy absorption leads to heat-induced changes in the Au film's transmittance. The change in transmittance leads to a large time-dependent thermo transmission signal on each photodetector of the balanced detector, see supplemental figure 1a.
(2) Circularly-polarized induced motion of charge in the Au causes birefringence. As a result, the polarization of the transmitted probe beam is rotated relative to the incident beam, which causes an asymmetry in the time-dependent signal measured by each detector.

The second effect is the signal of interest in a time-domain inverse Faraday effect measurement. The balanced photodetector outputs the sum of the voltages measured by the + and – photodiode. If the photodiodes are perfectly balanced, meaning the average intensity on each photodiode is exactly equal, and the thermo-transmission signals on each photodiode cancel each other. Supplemental figure 1d shows a TD-IFE measurement where the detectors are well-balanced. Supplemental figures 1b, 1c, 1e, and 1f show the results when the detector is not perfectly balanced.

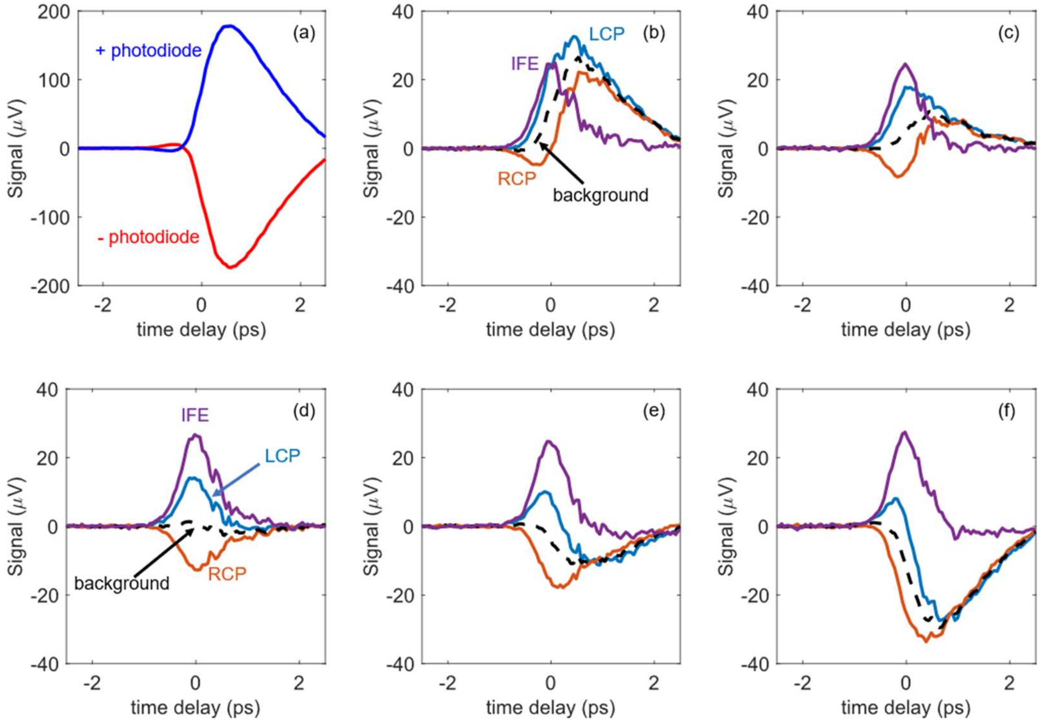

Supplemental Figure 1. Effect of time-dependent thermo-transmission background on the inverse Faraday effect signals. (a) Voltage measured by RF-lockin from each individual photodiode during an inverse Faraday measurement of a 20 nm Au thin-film. (b-f) Total voltage measured by the RF-lock in from the balanced detector (sum of the voltages from the + and – photodetector) for different levels of balancing. The blue curves are for LCP pump excitation, while the orange curves are experiments with RCP excitation. The DC (average) voltages on the + and – detector in each experiment are (b) 0.93 and 0.8 V, (c) 0.89 and 0.84 V, (d) 0.87 and 0.87 V, (e) 0.84 and 0.9 V, (f) 0.8 and 0.94 V. The purple curves are the IFE signal of interest, defined to be the difference in the LCP and RCP signals. The purple curve is not sensitive to small background levels of thermo transmission signals.

If the balanced photodiodes are imperfectly balanced, the signal recorded during a time domain inverse Faraday effect measurement includes a background thermo-transmission signal, see supplemental figure 1(b-f). The thermo-transmission background is shown as dashed black lines. This background leads to asymmetry in the measured IFE signals, which are shown in supplemental figure 1 with LCP and RCP labels. The background is independent of the pump beam's polarization, and so can be removed by defining the IFE signal as the difference in LCP and RCP measurements. As seen in Fig. 1, the presence of a background signal does not affect the IFE signal (purple curves). The raw TD-IFE signals shown in Figure 2 of the main-text include a small background that results in asymmetry of LCP and RCP light. This background does not affect the reported magnitude of the IFE effect, since it is subtracted out when considering the difference in the LCP vs. RCP measurements.
